# Supplementary material for: Low-cost anti-mycobacterial drug discovery using engineered E. coli
Source: Nat Commun. 2022 Jul 7;13:3905. doi: 10.1038/s41467-022-31570-3 (PMC9262897; doi:10.1038/s41467-022-31570-3)
Supplement: Supplementary file 1 — Supplementary information [file 41467_2022_31570_MOESM1_ESM.docx]

Supplementary information for:

**Low-cost anti-mycobacterial drug discovery using engineered *E. coli***

Nadine Bongaerts^1,2^, Zainab Edoo^3^, Ayan A. Abukar^1,2^, Xiaohu Song^1,2^, Sebastián Sosa Carrillo^1,4^, Sarah Haggenmueller^1,2^, Juline Savigny^1,2^, Sophie Gontier^1,2^, Ariel B. Lindner^1,2,^* & Edwin H. Wintermute^1,2^*

^1^ Université Paris Cité, Inserm, System Engineering and Evolution Dynamics, Paris, France

^2^ CRI, Paris, France

^3^ Sorbonne Université, Université Paris Cité, Inserm, Centre de Recherche des Cordeliers (CRC), Paris, France

^4^ Current address: Institut Pasteur, Inria de Paris, Université Paris Cité, InBio, Paris, France

* Corresponding authors: ariel.lindner@inserm.fr, jake.wintermute@cri-paris.org

Subject Terms: Synthetic biology; Chemical genetics; Bacterial infection; High-throughput screening; Assay systems

Included in this file:

[Supplementary Results 1](#_Toc106881602)

[Construction of an efflux-deficient TESEC host with minimal growth perturbation 1](#_Toc106881603)

[Evaluation of anti-mycobacterial activity of benazepril by spot plating 2](#_Toc106881604)

[Validation of an in vitro coupled assay for inhibition of Alr with benazepril 2](#_Toc106881605)

[Activity of hits from Asd and DapB screens against whole-cell *M. smegmatis* 3](#_Toc106881606)

[Effect of efflux activity on *E. coli* sensitivity to hit compounds 3](#_Toc106881607)

[Strains and plasmids 6](#_Toc106881608)

[Supplementary References 7](#_Toc106881609)

Supplementary Results

Construction of an efflux-deficient TESEC host with minimal growth perturbation

The TolC outer membrane channel is required for the function of many efflux systems that contribute to antibiotic resistance in *E. coli*^1^. While not all efflux activity is TolC-dependent, most clinically relevant forms of resistance are attributed to TolC and the many inner membrane permeases with which it interacts^2^.

Deletion mutants of *tolC* suffer from reduced growth rates and abnormal cell morphology. Vega and Young showed that these defects were caused by an accumulation of enterobactin in the periplasm and could be minimized by a compensating deletion in the isochorismate synthase *entC*^3^.

|  |
| --- |
| **Supplementary Figure 1 \| Deletion of *entC* partially restores growth and morphology defects of the efflux-deficient *tolC* deletion mutant.** **a,** The indicated deletions were introduced to the TESEC parent strain BW25113^4^. Overnight cultures of cells were diluted to 0.001 in defined medium and dispensed at 180 μL into 96-well, flat-bottom microplates. Cells were grown at 37 °C with shaking in a TECAN Infinite plate reader. Individual growth traces are shown for 3 biological replicates. **b,** Phase-contrast microscopy images (63x magnification) of strains with the indicated deletions. Cells were grown to saturation in defined medium. Scale bar is 10 μm. Images are representative of 3 biological replicates. Source data are provided as a Source Data file. |

To increase drug sensitivity while minimizing other phenotypic perturbations, we introduced the *tolC* and *entC* deletions in the TESEC Host (Supplementary Fig. 1). Deletion of *tolC* was associated with an extended lag phase but no reduction in growth rate or saturation density (Supplementary Fig. 1a). Cell morphology of the Δ*tolC* strain was elongated and aberrant as evaluated by phase microscopy (Supplementary Fig. 1b). Introduction of the compensating *entC* deletion in restored a wild-type rod shape and reduced, but did not eliminate, the extended lag phase.

Evaluation of anti-mycobacterial activity of benazepril by spot plating

We sought to characterize the activity of benazepril as a bactericidal in addition to its effect as a bacteriostatic described in the main text. Standard serial dilutions and spot plating *of M. smegmatis* mc^2^155 showed significant killing caused by both DCS and benazepril in the millimolar range (Supplementary Fig. 2).

|  | **Supplementary Figure 2 \| Benazepril and DCS show bactericidal activity near their growth-inhibitory concentrations.** *M. smegmatis* mc^2^155 cultures in Middlebrook 7H9 medium (Sigma M0178) were exposed to DCS (0-0.5 mM) or benazepril (0-4.5 mM) for 48 hours. Serial dilutions were prepared of the bacterial cultures and spotted as 1 μL aliquots onto Middlebrook 7H9 agar plates. A photo was taken after 48 hours of incubation at 37 °C. The image is representative of three independent experiments. |
| --- | --- |

Validation of an in vitro coupled assay for inhibition of Alr with benazepril

Our in vitro assay for Alr activity relies on a coupled reaction to alanine dehydrogenase^5^. Metabolic inhibitors, particularly substrate analogs, may act on multiple targets within a metabolic pathway if pathway intermediates are structurally similar. DCS, for example, acts both on Alr and the downstream D-alanine:D-alanine ligase^6^.

We therefore sought to confirm that benazepril does not inhibit L-alanine dehydrogenase directly. Neither DCS nor benazepril was active against L-alanine dehydrogenase under conditions similar to the full Alr assay (Supplementary Fig. 3).

| 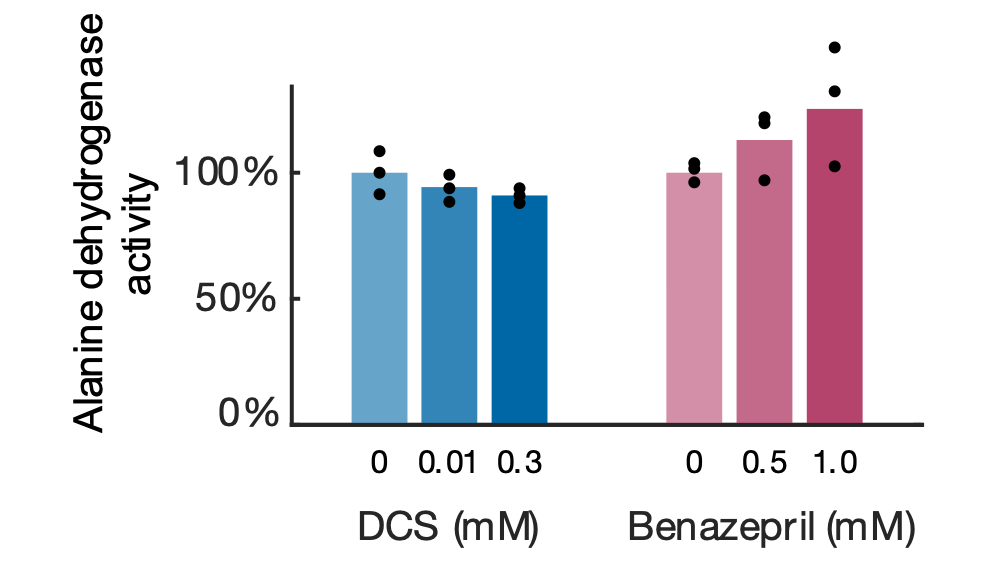 | **Supplementary Figure 3 \| Alr inhibitors do not affect the second enzyme of the in vitro coupled Alr assay.** L-alanine dehydrogenase from *Bacillus subtilis* (Merck A7653) was prepared at 50 U/μL in reaction buffer matching the Alr inhibition assays (20 mM Tris, 100 mM NaCl, pH 8.0, 20 mM NAD+). Addition of 5 mM L-alanine and benazepril or DCS at the indicated concentrations did not significantly reduce the rate of NADH production, as measured by time-course fluorescence measurements. Data are presented as the mean and individual data points for 3 biological replicates. Source data are provided as a Source Data file. |
| --- | --- |

Activity of hits from Asd and DapB screens against whole-cell *M. smegmatis*

To establish the scalability of the TESEC system, we extended the screen to additional enzymatic targets beyond Alr and identified additional hit compounds. For the TESEC strain expressing *Mtb* Asd, we identified diethylstilbestrol, bromhexine, deoxycorticosterone and riluzole as potential targeted inhibitors. For the DapB strain, we similarly identified pentamidine.

Following the protocol described for benazepril, we tested the anti-mycobaterial activity of the extended hit compounds. Only pentamidine was found to inhibit the growth of *M. smegmatis* with effective concentrations in the micromolar range (Supplementary Fig. 4). Pentamidine is an antimicrobial and anti-fungal used in the treatment of trypanosomiasis and pneumocystis^7^. It's mechanism of action is not fully described, with proposed targets including DNA replication, nucleotide synthesis, RNA polymerase and ribosomal function^8^.

Effect of efflux activity on *E. coli* sensitivity to hit compounds

In order for a compound to be identified through TESEC screening, it must first pass through the microbial membrane. By eliminating the TolC efflux system, we sought to increase the number of compounds that would pass this barrier. A consequence of this is that TESEC assays can detect compounds that are effluxed from wild-type cells and therefore lack whole-cell antibacterial activity.

| 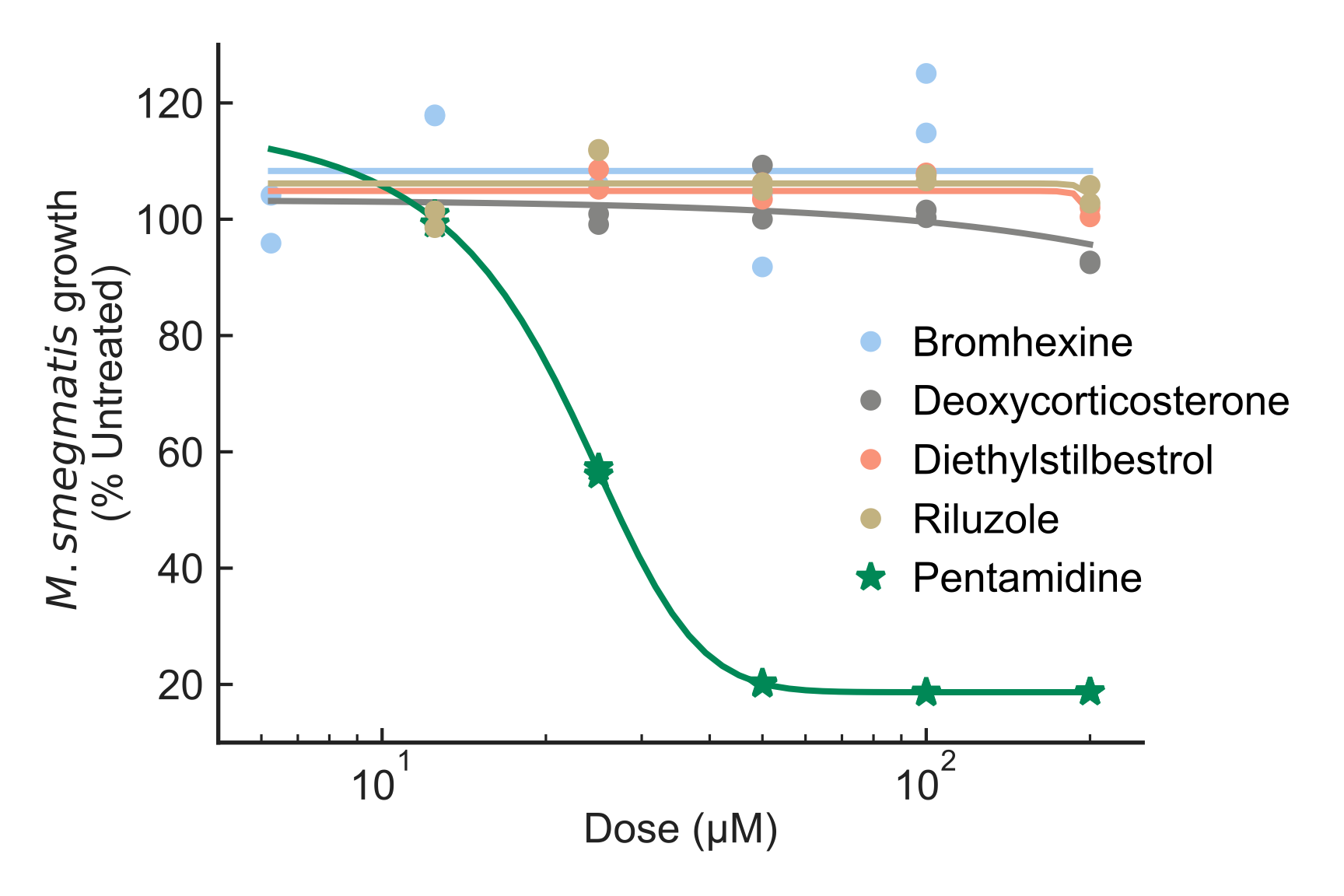 | **Supplementary Figure 4 \| Activity of the extended hit compounds in *M. smegmatis*.** Overnight cultures were diluted 1:100 in tryptic soy broth (TSB) supplemented with 0.5% Tween 80 and drugs at the indicated concentrations. Growth was measured by OD after 48 hours. Individual growth measurements are plotted, with at least two biological replicates for each series. Lines indicate the best-fit logistic regression. Source data are provided as a Source Data file. |
| --- | --- |

To characterize the relationship between efflux systems and drug sensitivity, we constructed a series of *E. coli* deletion mutants (Supplementary Fig. 5). In each mutant, we quantified the sensitivity to hit compounds from our assays targeting Alr (benazepril), Asd (Diethylstilbestrol, Bromhexine, Deoxycorticosterone, Riluzole) and DapB (Pentamidine).

With the exception of deoxycorticosterone, all hit compounds showed significantly increased sensitivity in the Δ*tolC* Δ*entC* background. This is consistent with the described role of TolC as an outer membrane channel coupling with multiple major pumping systems^1^. Deletion of other efflux activities had limited impact, indicating that they were not essential for pumping these particular compounds.

For four of the six hit compounds, loss of TolC efflux activity was essential for revealing the antibiotic activity. This highlights the capacity of the TESEC assay to reveal previously undescribed activities, as well as to a key limitation of the assay in identifying compounds that may lack whole-cell activity in efflux positive cells. Future work may expand TESEC assays to include panels of efflux mutants, using the system to characterize the efflux mechanism of specific hits.

| 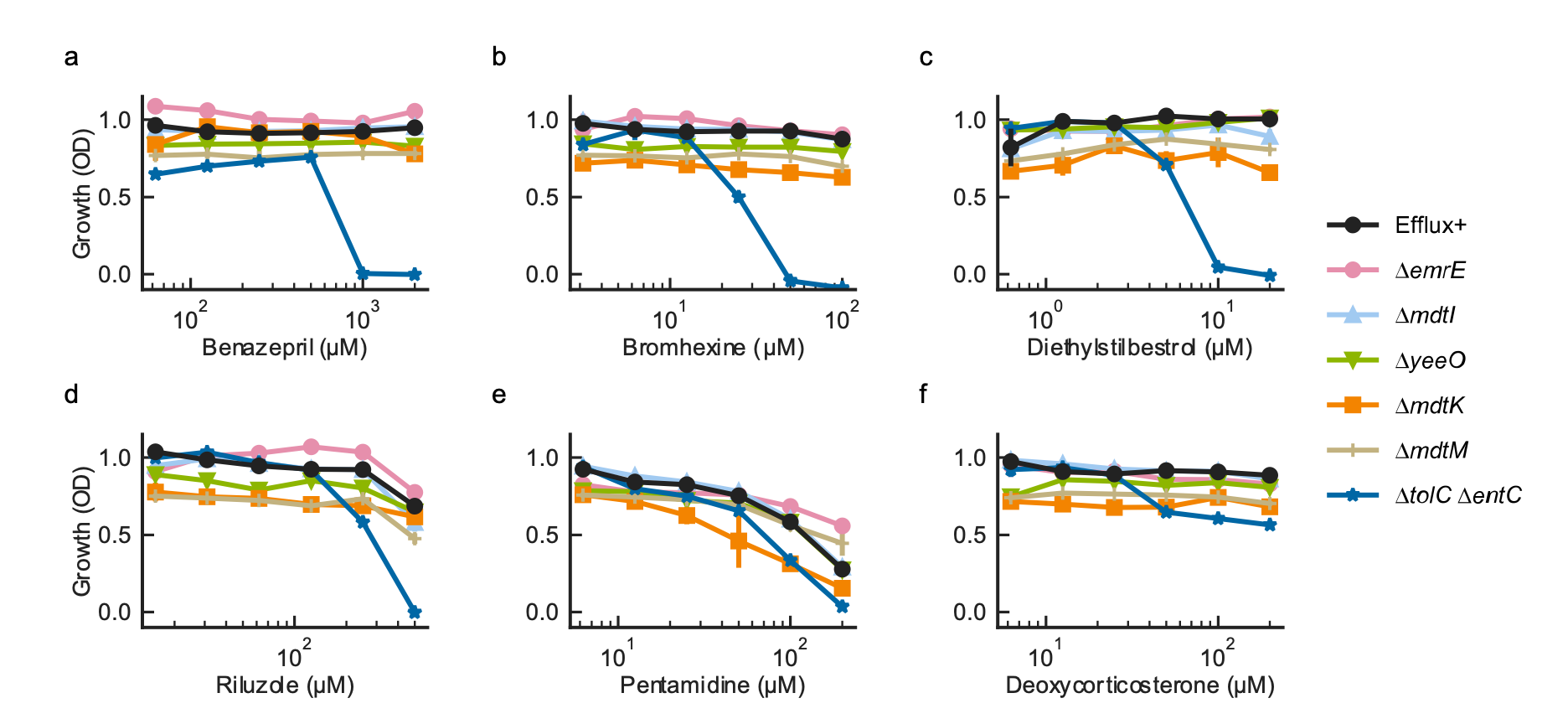 |
| --- |
| **Supplementary Figure 5 \| Effect of efflux pump deletions on the activity of TESEC hit compounds.** *E. coli* of either the TESEC parent strain, or carrying the indicated deletions, were cultured overnight and diluted 1:100 in M9-based medium with a dilution series of drugs at the indicated concentrations. Growth was measured by OD after 24 hours. Data is presented as the mean and CI_95_ of 3 biological replicates. |

Strains and plasmids

| Strain name | Parent | Relevant genotype | Source |
| --- | --- | --- | --- |
| BW25113 |  | *Δ(araD-araB)567 ΔlacZ4787(::rrnB-3) λ, rph-1 Δ(rhaD-rhaB)568 hsdR514* | ^4^ |
| TESEC Host | BW25113 | *ΔaraC, ΔtolC, ΔentC* | This study |
| TESEC Host Alr- | BW25113 | *ΔaraC, ΔtolC, ΔentC, Δalr, ΔdadX* | This study |
| TESEC Host Alr- Efflux+ | BW25113 | *ΔaraC, Δalr, ΔdadX* | This study |
| TolC+ Control | BW25113 | *ΔaraC, ΔentC* | This study |
| Ent+ Control | BW25113 | *ΔaraC, ΔtolC* | This study |
| TolC+ Ent+ Control | BW25113 | *ΔaraC, ΔtolC, ΔentC* | This study |
| TESEC Host asd- | BW25113 | *ΔaraC, ΔtolC, ΔentC, Δasd* | This study |
| TESEC Host cysH- | BW25113 | *ΔaraC, ΔtolC, ΔentC, ΔcysH* | This study |
| TESEC Host dapB- | BW25113 | *ΔaraC, ΔtolC, ΔentC, ΔdapB* | This study |
| TESEC Host trpD- | BW25113 | *ΔaraC, ΔtolC, ΔentC, ΔtrpD* | This study |
| Efflux+ | BW25113 | *ΔaraC* | This study |
| Δ*tolC,* Δ*entC* | BW25113 | *ΔaraC, ΔtolC, ΔentC* | This study |
| Δ*emrE* | BW25113 | *ΔaraC, ΔemrE* | This study |
| Δ*mdtI* | BW25113 | *ΔaraC, ΔmdtI* | This study |
| Δ*yeeO* | BW25113 | *ΔaraC, ΔyeeO* | This study |
| Δ*mdtK* | BW25113 | *ΔaraC, ΔmdtK* | This study |
| Δ*mdtM* | BW25113 | *ΔaraC, ΔmdtM* | This study |
| Supplementary Table 1 \| *E. coli* host strains used in this study. | | | |

|  | Template | Copy number | Marker | Expression Construct | Source |
| --- | --- | --- | --- | --- | --- |
| pNB101 | pRD123^9^ | Low (pSC101) | KanR | pBAD - AraC | This study |
| pNB201 | pRD131 | High (ColE1) | AmpR | pBAD - *Mtb* Alr | This study |
| pNB202 | pRD131 | High (ColE1) | AmpR | pBAD - mEGFP | This study |
| pNB203 | pRD131 | High (ColE1) | AmpR | pBAD - mEGFP - *Mtb* Alr | This study |
| pNB204 | pRD131 | High (ColE1) | AmpR | pBAD - 6xHis - *Mtb* Alr | This study |
| pNB205 | pRD131 | High (ColE1) | AmpR | pBAD - *Mtb* asd | This study |
| pNB206 | pRD131 | High (ColE1) | AmpR | pBAD - *Mtb* cysH | This study |
| pNB207 | pRD131 | High (ColE1) | AmpR | pBAD - *Mtb* dapB | This study |
| pNB208 | pRD131 | High (ColE1) | AmpR | pBAD - *Mtb* trpD | This study |
| Supplementary Table 2 \| Plasmids used in this study. | | | | | |

| Strain | Host | Plasmids |
| --- | --- | --- |
| TESEC *Mtb* Alr | TESEC Host Alr- | pNB101, pNB201 |
| TESEC *Mtb* Alr Efflux+ | TESEC Host Alr- Efflux+ | pNB101, pNB201 |
| TESEC *Mtb* Alr Purification | TESEC Host Alr- | pNB101, pNB204 |
| TESEC Alr+ Wild-Type Control | TESEC Host | pNB101, pNB202 |
| TESEC GFP-tagged *Mtb* Alr | TESEC Host Alr- | pNB101, pNB203 |
| TESEC *Mtb* Asd | TESEC Host asd- | pNB101, pNB205 |
| TESEC *Mtb* CysH | TESEC Host cysH- | pNB101, pNB206 |
| TESEC *Mtb* DapB | TESEC Host dapB- | pNB101, pNB207 |
| TESEC *Mtb* TrpD | TESEC Host trpD- | pNB101, pNB208 |
| Supplementary Table 3 \| TESEC strains created for this study | | |

| Strain name | Source |
| --- | --- |
| *Mycobacterium smegmatis* mc2155 | ATCC 700084 |
| *Mycobacterium tuberculosis* H37Rv | ATCC 25618 |
| Supplementary Table 4 \| Mycobacterial strains used in this study. | |

Supplementary References

1. Li, X.-Z. & Nikaido, H. Antimicrobial Drug Efflux Pumps in Escherichia coli. in *Efflux-Mediated Antimicrobial Resistance in Bacteria: Mechanisms, Regulation and Clinical Implications* (eds. Li, X.-Z., Elkins, C. A. & Zgurskaya, H. I.) 219–259 (Springer International Publishing, 2016).

2. Slipski, C. J., Zhanel, G. G. & Bay, D. C. Biocide Selective TolC-Independent Efflux Pumps in Enterobacteriaceae. *J Membrane Biol* **251**, 15–33 (2018).

3. Vega, D. E. & Young, K. D. Accumulation of periplasmic enterobactin impairs the growth and morphology of *Escherichia coli* tolC mutants. *Molecular Microbiology* **91**, 508–521 (2014).

4. Baba, T. *et al.* Construction of *Escherichia coli* K‐12 in‐frame, single‐gene knockout mutants: the Keio collection. *Mol Syst Biol* **2**, (2006).

5. Bergmeyer, H. U., Williamson, D. H. & Gawehn, K. *Methods of enzymatic analysis*. (Verlag Chemie, 1974).

6. Prosser, G. A. & de Carvalho, L. P. S. Metabolomics Reveal d-Alanine: d-Alanine Ligase As the Target of d-Cycloserine in *Mycobacterium tuberculosis*. *ACS Medicinal Chemistry Letters* **4**, 1233–1237 (2013).

7. Lemke, T. L. & Williams, D. A. *Foye’s Principles of Medicinal Chemistry*. (Lippincott Williams & Wilkins, 2008).

8. Bennett, J. E., Dolin, R. & Blaser, M. J. *Mandell, Douglas, and Bennett’s Principles and Practice of Infectious Diseases*. (Elsevier, 2019).

9. Daniel, R., Rubens, J. R., Sarpeshkar, R. & Lu, T. K. Synthetic analog computation in living cells. *Nature* **497**, 619–623 (2013).
